# Supplementary material for: Dominant ionic currents in rabbit ventricular action potential dynamics
Source: PLoS One. 2025 Jul 30;20(7):e0328261. doi: 10.1371/journal.pone.0328261 (PMC12310051; doi:10.1371/journal.pone.0328261)
Supplement: Attachment — Submitted filename: Response_to_Reviewer_1.pdf [file pone.0328261.s001.pdf]

Response to Reviewer 1

Journal: PLOS One
Manuscript ID: PONE-D-25-13282
Title: Dominant ionic currents in rabbit ventricular action potential dynamics
Authors: Yang, Gao, Smith, Simitev
Date: June 1, 2025

Dear Reviewer 1,

This paper presents a detailed sensitivity analysis of a rabbit model of ventricular cells. Similar investigations have been published in the past, but I liked how this study was conducted. The rationale for studying this specific rabbit model was convincing, considering the experimental variability observed in a cited paper. The methods are sound and sufficiently self-contained, and the results are well presented.

Thank you for you comments! We are pleased to see that you appreciate the importance of our work.

Other sensitivity analyses are summarized in Table 1 but there is no equivalent global sensitivity analysis for this type and species of cardiomyocyte.

We have revised our manuscript to address your comments and in the process included the following substantially new material:

- One new figure (Figure 1) has been added to demonstrate steady-state at the number of pre-pacing beats considered.
- Figures 2 and 5 have been substantially to include additional new data.
- A new section 3.4 has been added to extend the analysis to intracellular calcium biomarkers.

- Three additional references have been included to reflect relevant recent literature.

These additions required a significant number of new simulations to be performed. In particular, we repeated the full analysis for two additional stimulus amplitudes and performed a further set of simulations for calcium biomarker measurements. The main conclusions of the original manuscript remain unchanged.

All changes in the revised manuscript are marked in red. Please, find below detailed responses to your comments.

R1.Q1

The weights w_k were set to 1, but the biomarkers vary a lot in magnitude (B in particular). Would the results be different if B was divided by 1000? I think that based on (5a) it should not be different, but I felt that mentioning this invariance near line 193 would be reassuring.

A.R1.Q1

- Yes, The results for Sobol indices will remain the same if bulk B is divided by 1000. This is because all biomarker values were z-score standardised before performing the Sobol analysis. This is something we forgot to mention in our manuscript.
- **We have now stated this in the first paragraph of section 2.2 and modified this section as appropriate**. This does not affect the analysis.

R1.Q2

2) Besides the effect of pacing frequency, an issue with single cell models is the effect of stimulus strength. It would be useful to (1) document the stimulus current applied as compared to the stimulus threshold (minimum to elicit an AP or a sequence of APs), and (2) vary the stimulus between 1x and 2x the threshold and measure the effect on the biomarkers, just to see how it compares to the amplitude of the effect of the p_i.

A.R1.Q2

- We have investigated the effect of the stimulus strength. In paricular, we have repeated the full Sobol sensitivity analysis at two different stimulus amplitudes (14 A/F and 19 A/F), in addition to the results at 9.5 A/F originally reported. The threshold value for excitation is 9.4 A/F.

- We find that, varying the stimulus amplitude primarily affects the region of normal response (see updated Fig 2), but does not significantly affect the ranking of parameters (see updated Fig 5).
- **Results are shown in Fig 2 and Fig 5 which are now both significantly extended with new data. Summaries of this investigation are included in the last paragraph of section 3.1 and below equation (9) in section 3.3.**

R1.Q3

3) Why did you not use p_{Na} values below 1? Sodium blockers are common antiarrhythmic drugs. Is it in any way related to the choice of stimulus current? The comment at line 390 makes me wonder. Some comments in the discussion/conclusion would be helpful.

A.R1.Q3

- The range of values of p_{Na} that elicit normal response is determined as detailed in Section 3.1.
- However, when addressing your previous comment, we found that at larger amplitudes the range of p_{Na} can be extended below 1 as you suspected. This is shown in Fig 2(c). The extended range does not significantly change the ranking order of parameters as shown in Fig 5.
- **We have replaced panel (c) of Figure 2 to show the range of p_{Na}. This panelk (c) was previously showing the analysis for p_{Cap}. Other revisions to the manuscript are as already stated in the response R1.Q2**.

R1.Q4

4) About the Clb current, I'm wondering how long (number of beats) it takes to have an effect. Is it a slow drift that takes minutes or more?

A.R1.Q4

- We find that at 1000 beats the Clb current has already settled.
- We have investigated the effect of increasing the number of beats in the train as shown in a newly added figure – Figure 1. All AP biomarkers remain virtually unchanged beyond 500 beats.

- The Sobol sensitivity analysis becomes substantially more computationally expensive for large number of beats, so in our “standard” setup we used 1000.

- **We have added a new Figure 1, and discussion in the first paragraph of section 3.**

R1.M1

1) Table 3: I think that what you call resting potential is generally called maximal diastolic potential because it's measured during pacing. This could be mentioned in the table.

A.R1.M1

- Thanks. We have added this term in Table 3.

R1.M2

2) Eq.(5b): Why is the first summation over all i != j and not only the values of j such that i < j ? Does S_{ii} even exist?

A.R1.M2

- Equation (5b) is correct. The sums are written so that (a) repeated subscripts are excluded because self-interactions are meaningless, and (b) combinations that have appeared before are also excluded to avoid double counting. To help convince the Reviewer, we include below an explicit example of formula (5b) for a small number of variables with the disallowed terms crossed-out:

$$
\begin{aligned}
S_{T_3} = S_3 + \big(S_{31} + S_{32} + \xcancel{S_{33}} + S_{34}\big) + \big(\xcancel{S_{311}} + S_{312} + \xcancel{S_{313}} + S_{314} + \xcancel{S_{321}} + \xcancel{S_{322}} \\
+ \xcancel{S_{323}} + S_{324} + \xcancel{S_{331}} + \xcancel{S_{332}} + \xcancel{S_{333}} + \xcancel{S_{334}} + \xcancel{S_{341}} + \xcancel{S_{342}} + \xcancel{S_{343}} + \xcancel{S_{344}}\big) + S_{1234}
\end{aligned}
$$

- **No changes are made**.

R1.M3

3) line 189: Should it be "parameters p_i" considering (5c)?

A.R1.M3

- Yes, this must be "parameters p_i", indeed. **We have corrected this**.

R1.M4

4) line 191: "the the"

A.R1.M4

- **Yes, we have corrected this**.

R1.M5

5) lines 224-226: A reference here would be useful.

A.R1.M5

- **Done.**
- The reference (Qu et al (2014), Nonlinear and stochastic dynamics in the heart) describes both alternans and more complex chaotic responses and **is now cited at these lines**.

R1.M6

6) section 3.2: Maybe I missed it, but is the random distribution of parameters p_i uniform over the range given in Table 4?

A.R1.M6

- Yes. In fact, p_i are sampled by drawing multidimentional Sobol low-discrepancy sequences, which are designed to fill space better than random samples from a multivariate uniform distribution.
- **This is stated at the beginning of the penultimimate paragraph of section 2.2 immediately after equation (5.b), and now highlighted.**

R1.M7

7) Table 4: Why did you not use p_{Na} values below 1? Sodium blockers are common antiarrhythmic drugs.

A.R1.M7

- Please, see response to your query R1.Q3, above.

R1.M8

8) Fig. 3: "of the the background..."

A.R1.M8

- Corrected. Thank you!

R1.M9

9) ref 13: incomplete name of the second author

A.R1.M9

- Corrected. Thank you!

We hope that we have been able to address the concerns of the Reviewer and that they can now recommend our manuscript for publication.

With best regards,
The Authors
