# Supplementary material for: Dominant ionic currents in rabbit ventricular action potential dynamics
Source: PLoS One. 2025 Jul 30;20(7):e0328261. doi: 10.1371/journal.pone.0328261 (PMC12310051; doi:10.1371/journal.pone.0328261)
Supplement: Attachment — Submitted filename: Response_to_Reviewer_2_round_2.pdf [file pone.0328261.s002.pdf]

Response to Reviewer 2

Journal: PLOS One
Manuscript ID: PONE-D-25-13282
Title: Dominant ionic currents in rabbit ventricular action potential dynamics
Authors: Yang, Gao, Smith, Simitev
Date: June 19, 2025

Dear Reviewer 2,

Thank you for your further comments. We have revised our manuscript to address them. Changes in the revised manuscript are marked in red.

R2.Q1

... I understand that p_i factors are considered in Equation 1, which is used to calculate the membrane potential once the currents are known, but p_i factors are not considered when the derivative of the ionic concentrations are calculated taking into account the corresponding ionic currents. Equations 2- 6 of Shannon et al. 2004 (DOI: 0006-3495/04/11/3351/21) describe the derivative of ionic concentrations. The derivative of the ionic concentrations depend on the magnitude of the currents, which are modulated by p_i. Therefore, p_i must scale their respective currents in these equations as well.

A.R2.Q1

- **The factors p_i are properly included in both the membrane potential equation, as well as in the ionic concentration equations as appropriate.** We misunderstood your first-round query on this point. Apologies for the confusion!

- **We have now modified equations 1 and the paragraph directly underneath to clarify this explicitly.**

R2.Q2

... biomarkers appear in the same graph using a logarithmic scale ranging from 1 to 20000, which makes difficult to see small differences. Moreover, the magnitude of $[Ca^{2+}]_i$ *is not included. ....*

A.R2.Q2

- We have modified Figure 1 to plot the biomarkers normalized to their values for the longest train (2000 APs). Convergence to 0% indicates steady state. **We have removed the logarithmic scale which is now not needed**.

- **We have added** $[Ca^{2+}]_i$ **biomarkers to the Figure 1.**

- Few and rare random fluctuations occur occasionally due to roundoff numerical error accumulating in long train-simulations. These fluctuations occur in few biomarkers only, they are less than 2%, and we do not consider them to be significant.

- **We have added a remark to this effect at the beginning of Section 3.**

Thank you again for your comments.

With best regards,
The Authors
